# Promotion factors of emergency nurses’ post-traumatic growth during the COVID-19 pandemic in Shanghai: a qualitative study

**DOI:** 10.1186/s12912-023-01452-0

**Published:** 2023-09-01

**Authors:** Jinxia Jiang, Peng Han, Yue Liu, Qian Wu, Haiyan Shao, Xia Duan, Yan Shi

**Affiliations:** 1 Emergency Department, Shanghai Tenth People’s Hospital, School of Medicine, Tongji University, Shanghai, 200072 China; 2 Nursing Department, Shanghai Tenth People’s Hospital, School of Medicine, Tongji University, Shanghai, 200072 China; 3 Nursing Department, Shanghai First Maternity and Infant Hospital, School of Medicine, Tongji University, Shanghai, 201204 China

**Keywords:** Post-traumatic growth, Promotion factors, Emergency nurses, COVID-19, Psychological experience, Qualitative study, China

## Abstract

**Background:**

Since March 2022, Shanghai, China, has experienced a severe wave of SARS-CoV-2 transmission caused by the Omicron variant strain. The pandemic has severely constrained the local healthcare system. After treating critically ill COVID-19 patients, emergency nurses may experience some positive changes due to new insights or gains in their work, even if they have had traumatic experiences. This study aimed to explore the promoting factors of emergency nurses’ post-traumatic growth during the COVID-19 pandemic in Shanghai. We hoped to provide a new perspective and theoretical basis for intervening in and promoting the psychological rehabilitation of medical staff after traumatic circumstances.

**Methods:**

This study employed a qualitative design based on the phenomenological approach. 18 participants from the emergency department of a third-level class-A hospital in Shanghai who participated in treating COVID-19 patients were enrolled using purposive sampling. Data collection was through in-depth and semi-structured interviews and continued until reaching data saturation. The seven-step Colaizzi process was used for data analysis.

**Results:**

The investigation uncovered two themes and six subthemes. Internal factors contained self-affirmation, deliberate rumination, and cognitive restructuring, which constituted attitudes and behaviours that participants could subjectively determine. External factors included social support, transformational leadership, and role modelling, which constituted factors influenced by others or the environment.

**Conclusions:**

The promoting factors of PTG of emergency nurses originated from different sources such as individuals, organizations, and society. In addition to good psychological adjustment of the individual, society, hospitals, and nursing managers should focus on establishing supportive PTG strategies. The ultimate purpose is to improve the retention rate and career growth of nurses.

## Background

In March 2020, the World Health Organization declared the severe acute respiratory syndrome coronavirus 2 (SARS-CoV-2) pandemic that caused the 2019 coronavirus disease (COVID-19) as a Public Health Emergency of International Concern, characterizing it as a pandemic [1]. The Omicron strain is currently the dominant pandemic strain worldwide, with a shorter incubation period, stronger infectivity, and faster transmission speed. Omicron’s complex and hidden transmission characteristics make it difficult to prevent and control. Since March 2022, Shanghai, China, has experienced a severe wave of SARS-CoV-2 transmission caused by the Omicron variant strain, and nearly 650,000 people have been infected [2].

The Chinese government thusly summoned more than 38,000 medical staff from 15 provinces to Shanghai to participate in the relief efforts [3]. Many medical resources have been mobilized, and more than 100 designated hospitals and mobile cabin hospitals with over 183,000 beds have been set up to manage the massive epidemic impact [3].

Nurses are critical in the fight against COVID-19 and undertake complex, intense, and difficult work, especially among front-line emergency nurses. Specifically, in addition to emergency nursing work, emergency nurses have also been required to screen COVID-19-positive patients and conduct epidemiological investigations and transfer of patients and close contacts, environmental disinfection, and sampling of the department [4]. The basic nursing work that was originally undertaken by nursing assistants has also been completed by emergency nurses. In the emergency department, some nurses were dispatched to work in designated hospitals and mobile cabin hospitals, which led to a shortage of human resources. Moreover, nurses from other departments could not effectively alleviate the surge in workload in the emergency department due to their lack of first aid experience. In addition, the patients’ worsening conditions, the risk of infection, poor communication at work, and the uncertainty caused by the pandemic contributed to their stress, which deteriorated nurses’ physical and psychological health. Nurses’ physical and mental health at work is closely related to the quality of patient care [5].

Meanwhile, some studies have demonstrated that, compared with the general population, the trauma degree of nurses working in the COVID-19 front line is higher, has more sources, and mainly manifests as depression, anxiety, fear, and other negative emotions [6]. Specifically, in the COVID-19 context, the superposition of stressors makes nurses more susceptible to psychological trauma in high-exposure and high-risk working environments [7].

Psychological trauma can be self-healing, while a significant degree of trauma may cause anxiety and depression, and severe cases may cause post-traumatic stress disorder (PTSD). Thus, these individuals are at high risk of secondary traumatic stress, which seriously threatens their mental health and quality of life [8]. However, people’s psychological perceptions of experiencing trauma are not only negative, and recently, some scholars have tried to understand people’s subjective feelings other than secondary traumatic stress from a positive perspective. The positive psychological changes that occur after an individual experiences traumatic events are known as post-traumatic growth (PTG). PTG refers to a person’s ability to grow because of trauma [9]. After treating critically ill COVID-19 patients, nurses may experience some positive changes due to new insights or gains in their work, even if they have had traumatic experiences. PTG helps them to reflect on their experience, which benefits their career growth and general satisfaction with life.

Undeniably, nurses can still dispel pain and make progress after experiencing trauma, which is immensely helpful for accumulating experience and achieving career growth. However, research on which factors promote their PTG is still lacking, especially in the COVID-19 pandemic context. Emergency nurses play an indispensable role in front-line work; simultaneously, however, they constitute a population at high risk of trauma. It is thus of interest to study their PTG amid highly stressful work. Therefore, this study selected emergency nurses during the COVID-19 pandemic in Shanghai as the study participants and used the phenomenological research method of qualitative research to explore the promoting factors of their PTG.

### Study aim

The aim of this study was to improve the understanding of the enabling factors that enable emergency nurses to cope with adversity in their nursing work and and to explore the role of these factors in working in the context of COVID-19. Considering the importance and lack of studies, the researchers attempted to provide a new perspective and theoretical basis for intervening in and promoting the psychological rehabilitation of medical staff after traumatic events.

## Methods

### Study design

We used phenomenological qualitative research and face-to-face semi-structured interviews to explore the subjective feelings and experiences of PTG among emergency nurses during the COVID-19 pandemic in Shanghai in March 2022, as well as the promotion factors of PTG. In qualitative research, phenomenological methods focus on describing common experiences shared by the entire population, which also helps researchers to engage with participants from an in-depth perspective and to understand their experiences [10].

### Participants and ethical considerations

Nurses in the emergency department of a third-grade class-A hospital (general hospital with the highest level of medical service qualification assessed by the Ministry of Health of China) in Shanghai who participated in treating COVID-19 patients from March to June 2022 were included in the study. Participants were selected via purposeful sampling, which is used to identify and select informative cases related to the phenomenon of interest when the sample size is small [11]. Inclusion criteria were as follows: (1) Emergency department nurses, (2) working for more than two months during the COVID-19 pandemic, and (3) engaged in the frontline treatment of COVID-19 patients. Exclusion criteria: Trainee nurses or probationers. Potential participants were contacted via e-mail. Ethical approval was obtained from the Institutional Review Committee of Shanghai Tenth People’s Hospital (Approval No. 22KN08). Permission was obtained from the participants to arrange one-on-one individual interviews, all nurses gave their written informed consent to participate in the study prior to the start of the interview. Face-to-face interviews were conducted in a separate and quiet room on the ward. All interviews were digitally recorded, pseudonymized and transcribed verbatim. We ensured that participants were aware of the purpose and process of the study and emphasized the privacy of the environment and the confidentiality of the data. Finally, 18 emergency nurses were interviewed for 875 min (mean, 48.61 min). The interviews also continued to reach data saturation, meaning no further data and new concepts were obtained on the topics of interest.

### Data collection

Semi-structured, in-depth, face-to-face interviews were conducted in August 2022. As shown in Table 1, we developed a semi-structured interview guide for data collection based on previous studies [12, 13]. Participants shared their traumatic experiences and coping processes, which were explored until they fully understood emerging themes. The guide commenced the interview with encouraging questions. If necessary, the interviewer would ask more in-depth and specific follow-up questions. It must be emphasized that the participants had no relationship with the researchers before the study began and were previously unknown to them. In addition, two emergency nurses were selected for a pre-interview prior to data collection to ensure the clarity of the questions and to identify any potential problems. The pre-interviews were treated as tests and were excluded from the analysis. Formal interviews lasted 40 to 60 min each, were audio recorded with permission, and participants’ responses, including nonverbal cues and body language during the interviews, were noted. Interviews were conducted by a group of two researchers and two research assistants. Each interview was transcribed verbatim by researchers and analysed concurrently.

**Table 1:** Interview guideline: Open questions

No.	Questions
1	What positive psychological experiences or changes have you had during the COVID-19 pandemic?
2	What are the reasons for your positive changes?
3	How have you managed the stress and difficulties of fighting the COVID-19 pandemic?
4	What has been touching and unforgettable about your work experiences during the COVID-19 pandemic?
5	Who are you most thankful for during the COVID-19 pandemic? Why is that?
6	What additional perceptions and experiences did you have?

### Data analysis

Audio recordings were transcribed verbatim and checked for accuracy by repeated listening within 24 h of the interviews. After the interview, the data were analysed separately and immediately by two researchers with skilled analysis experience. Interview data was analysed using Nvivo 12.0, a computer-assisted qualitative data management software. Colaizzi’s phenomenological seven-step method [14] was used for data analysis to complete the extraction of themes and sub-themes regarding the promoting factors of PTG among emergency nurses during the COVID-19 pandemic (see Table 2). Any disagreement between researchers was resolved by making decisions through discussion until a consensus was reached. The final transcribed data, as well as the extracted themes and sub-themes, were sent to the participants simultaneously, and all participants agreed to be contacted again. Feedback communication ensured that the extracted results reflected the participants’ actual views and that the investigators’ subjective perceptions did not bias the results.

**Table 2:** Colaizzi’s seven-step process for qualitative data analysis

No.	Data analysis step
1	All interviews were recorded and transcribed. Each transcript was carefully read several times.
2	Researchers re-read, highlighted, and extracted meaningful statements directly related to the perspectives and experiences that promoted PTG in emergency nurses.
3	Meanings from all significant statements were summarized.
4	Identified and organized the formulated meanings into theme clusters. The researchers compared the theme clusters with the original data several times to determine consistency.
5	Exhaustively described the investigated phenomenon of the experience of promoting PTG in emergency nurses.
6	Recognized similar subthemes, identified the basic structure, and obtained the main themes.
7	Returned to the participants to confirm the findings. The authors discussed their disagreements until a consensus was reached.

### Study rigor

To ensure the study’s dependability, the methods and analyses used were described in detail. Each researcher ensured credibility via a ‘blinded’ approach to the materials, the researchers fully considered contradictory data caused by the diversity in sampling, established relationships with the participants, persistent observation, searched for documents and evidence, analyzed negative cases, reflected on the credibility of the researchers. Validity was ensured by continuous triangulation among researchers regarding discordant texts. Peer information was used for verification. In addition, the data were re-evaluated by an expert in qualitative studies who was not involved in the study. Finally, for the transferability of the data, the sample and the data were described in detail.

## Results

**Fig. 1:**
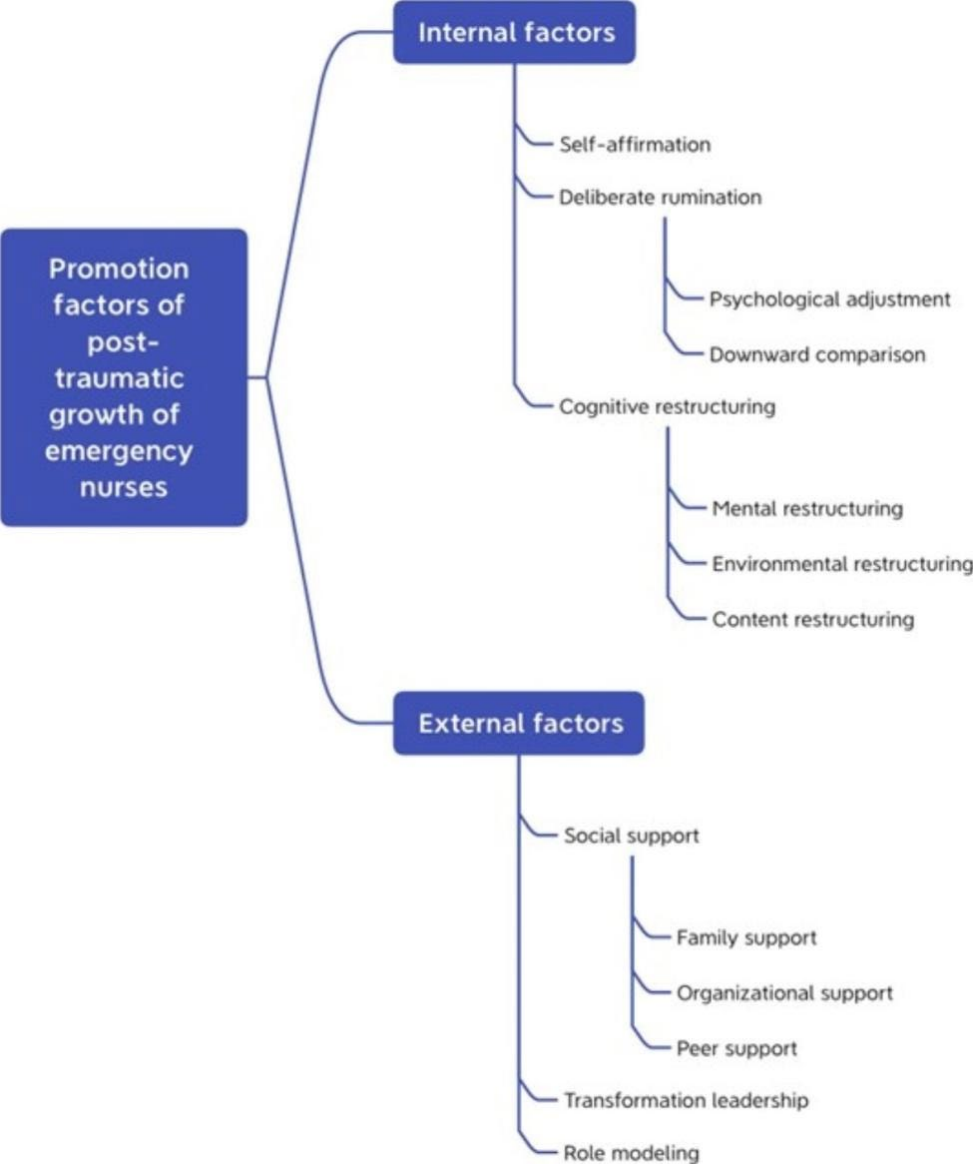
Themes and sub-themes categorized from data

A total of 18 participants (6 males and 12 females) were included in the study. Their average age was 30.72 years, and their average length of employment was 8.56 years. Regarding the level of previous nursing education, participants had either a university diploma (roughly equivalent to an associate degree, 11.1%), bachelor’s degree (83.3%), or postgraduate degree (5.6%). Most participants were unmarried (61.1%). Participants’ general characteristics were shown in Table 3. Using Colaizzi’s methodology, two main themes were extracted to describe the promotion factors of PTG of emergency nurses during the COVID-19 pandemic in Shanghai: (a) Internal factors, included the attitudes and behaviours that participants could subjectively determine among the promotion factors of PTG; and (b) External factors, included the part of the promotion factors of PTG in which the participant’s situation was influenced by others or environment. There were several different subthemes within each theme. Themes and sub-themes categorized from the data are presented in Table 4 (Fig. 1).

**Table 3:** Participants’ general characteristics

Characteristics	Mean (SD)	N (%)
**Sex**		
Male		6(33.3)
Female		12 (66.7)
**Age (years)**	30.7 (4.7)	
**Length of employment**	8.6(4.9)	
**Education Level**		
Diploma		2 (11.1)
Bachelor’s Degree		15(83.3)
Master’s degree		1(5.6)
**Marital status**		
Married		7 (38.9)
Single		11(61.1)

**Table 4:** Themes and sub-themes categorized from data

Themes	Definition	Subthemes
**Internal Factors**	This theme included the attitudes and behaviours that participants could subjectively determine among the promotion factors of PTG.	• **Self-affirmation** • **Deliberate Rumination** Psychological adjustment Downward comparison • **Cognitive Restructuring** Mental restructuring Environmental restructuring Content restructuring
**External Factors**	This theme included the part of the promotion factors of PTG in which the participant’s situation was influenced by others or environment.	• **Social Support** Family Support Organizational Support Peer Support • **Transformational Leadership** • **Role Modelling**

### Internal factors

#### Self-affirmation

The tradition of oriental philosophy considers humans as an integral part of nature. Some participants constantly affirmed themselves and viewed trauma and crisis from a positive perspective: ‘*I rely on a positive state of mind to succeed. It is better to maintain a good attitude in adversity, let nature take its course, and accept it. I think the working environment of the emergency department has created a strong spirit within me. No matter what difficulties I experience, I believe that every cloud has a silver lining*’ (Participant 11). Culture and beliefs governed individual experience and feelings and encouraged individuals to face trauma: ‘*I keep telling myself, trauma is a part of life, this is nothing, believe in yourself! (smiling)*’ (Participant 6). One participant viewed trauma as a necessity in life to motivate themself and to generate energy. ‘*No matter work, study, or life cannot be as calm as a lake, every setback will make me grow a little bit. We must learn to overcome uncertainty with certainty, just like fighting monsters in video games, and I believe I have the potential to do so*’ (Participant 13).

#### Deliberate rumination

Rumination is defined as the thoughts and behaviours that force individuals to focus on their emotional state, including intrusive and deliberate rumination. Intrusive rumination targets the individual’s negative perception of the traumatic event, whereas deliberate rumination refers to individuals actively and repeatedly re-examining and contemplating the traumatic events and the clues related to traumatic events. Studies have shown that an individual’s deliberate rumination is positively correlated with PTG [15]. Most participants expressed experiences of deliberate rumination, including psychological adjustment and downward comparison. ‘*Sometimes I look back on that experience and try to attain a balanced self*’ (Participant 2). The following participant illustrated the example of witnessing the negative experiences of others and their own rumination of downward comparison. ‘*After the middle school entrance examination last month, a boy quarreled with his parents and jumped from the 6th floor. He was sent to our hospital with no vital signs, and his parents cried beside the cold body… (shaking head), but the 14-year-old will never come back. I have seen so many deaths in the emergency department that I feel my experience is really insignificant compared to the pain others have experienced*’ (Participant 9).

#### Cognitive Restructuring

Understanding the order in which emotions are generated is of interest because it allows one to act to avoid generating unhelpful emotions or to adjust them after they have already formed. A technique that psychologists call cognitive restructuring (CR) is one of the most effective ways for people to regulate their emotions; this approach is also known as cognitive reframing or cognitive reappraisal [16]. CR includes mental, environmental, and content restructuring. Mental restructuring is conducted to change one’s mindset and choose a different perspective to re-examine trauma. ‘*Try to calm down. The pandemic made me re-examine myself. In fact, people grow during challenges*’ (Participant 1). Environmental restructuring involves transforming a negative situation into a positive one. ‘*I think of a story (smiling). A farmer keeps a donkey, one day the donkey fell into a well, after evaluation, the farmer decided to shovel dirt and bury the donkey in the well. At first, the donkey fiercely resisted, but when the strength of the soil continued to impact, the donkey began to change its strategy. Every time the soil fell off, it would shake the soil and then go up a step, shake the soil, go up a step, and so on. Finally, although the donkey was tortured and exhausted, it successfully escaped the well. I think that if animals can understand the truth, why can’t I?*’ (Participant 3). Content restructuring constitutes changing the meaning of the traumatic event itself to the individual. ‘*Despite the busy and chaotic work under the COVID-19 pandemic, my comprehensive ability has been further improved*’ (Participant 7).

### External factors

#### Social Support

Social support refers to the social resources provided by formal or informal support groups that are perceived subjectively and/or received objectively by individuals. External support from the government, healthcare organizations, nursing managers, colleagues, friends, and family could help nurses to improve their ability to adapt to stress and achieve psychological adjustment [17]. Among the elements extracted from the participants’ descriptions in this study, the composition of social support mainly included their family, organization, and peers.

#### Family support

All participants expressed strong family support, which could give them a significant spiritual boost during tough times. ‘*Although I have not been home for two months, I feel the warmth of my family every day, and my family is the source of power for me to keep going*’ (Participant 6). Chinese family kinship is relatively close compared with the West. Elders attach significant importance to their families and children, and correspondingly, children are more willing to care for and accompany their parents and grandparents. ‘*Although my hometown is thousands of miles away, my parents show their care for me through video calls and share their life with me. For me, it is good that my parents are healthy and safe (smiling)*’ (Participant 10).

#### Organizational support

Many nurses expressed that they received powerful organizational support that was helpful to maintain a positive mental state. ‘*The hospital department has given us a lot of support, such as daily work subsidies during the pandemic period, increased performance pay, etc., which makes me feel very pleased*’ (Participant 10). Chinese culture and health systems focus on active care and intervention. Some nurses mentioned meaningful activities carried out by hospitals and departments, and they achieved PTG under organizational care. ‘*Several music therapy and meditation workshops were held in the hospital, and I gained new skills and benefited a lot*’ (Participant 16). ‘*Some online work forums were arranged in the department, and everyone spoke freely and with open minds. After speaking out about a lot of things, the pressure was less*’ (Participant 8).

#### Peer support

Participants indicated that peer support had helped them during difficult times and that the traumatic experience had brought them closer together. ‘*Like-minded friends are really a good medicine, sometimes I feel that I can’t think of things, through good friends, I suddenly feel clear (chuckling)!*’ (Participant 4). The experience of facing difficulties together promoted close relationships between colleagues and helped each other to grow. ‘*My colleagues in our department are very nice, helping each other in difficult times, joking with each other under pressure, and many unpleasant things are forgotten (laughing)*’ (Participant 9).

### Transformational leadership

Transformational leadership is an effective leadership style in healthcare organizations. The significance of organizational commitment, employee engagement, job satisfaction, and its impact on patient safety and outcomes are widely recognized [18]. Most participants expressed the facilitation of PTG through a transformative leadership style. ‘*The director of our department took the lead and set an example, so our team cohesion was very strong. Although the work was very hard, the working atmosphere was particularly good*’ (Participant 5). ‘O*ur head nurse is an excellent leader, and she has been on the front line of treatment with us during the epidemic. I think we have no reason to be depressed or complain*’ (Participant 15).

### Role modelling

During the COVID-19 pandemic, participants believed that professional mentors, clinical experts, or senior colleagues often served as role models and that their behaviours were encouraging and appealing. Nurses were inspired to become strong to overcome difficulties. ‘*Our professional tutor, Dr. Wang, is really very good (thumbs up). During the pandemic, in addition to the daily work, she also kept busy with the management of environmental sampling and disinfection, epidemiological investigation, treatment, and transport of infected patients, etc., worked more than ten hours almost every day. She is 46 years old, but she is still so determined to work overtime, I really can’t do it (smiling and shaking head); so, whenever I flinch, I will think of her and feel that there is nothing to pass*’ (Participant 17).

## Discussion

COVID-19 epidemic has affected almost all areas of human life. What is clear is that trauma among nurses during the COVID-19 pandemic is pervasive, however, a variety of internal and external factors contribute to their recovery from traumatic events. This study was conducted to reveal the promoting factors of emergency nurses’ PTG during the COVID-19 pandemic in Shanghai. Individuals, organizations, and societies respond effectively and positively to reduce damage caused by trauma, recover, and grow from it. The results of some previous studies were consistent with our findings. Tomaszek et al. emphasized that personal qualities, optimism, altruism, and organizational influence could constitute positive influencing factors of PTG [19]. Cui’s investigative research suggested that self-confidence in frontline work, awareness of risk, psychological intervention or training during the epidemic and deliberate rumination were the main influencing factors of PTG of nurses [20]. Some studies have also revealed other results, such as marriage, religion, self-disclosure, past traumatic experiences and other promoting factors [21, 22]. Our findings provide a foundation for further research on PTG among medical staff while discussing how an individual or team PTG can be influenced and improved by feasible support mechanisms or intervention strategies. The following is the specific discussion based on the study findings.

Internal factors were the first theme to emerge from this study and contained the attitudes and behaviours that participants can subjectively determine and proceed to aid the realization of PTG. A self-affirmation is an act that demonstrates one’s adequacy. It fosters an approach orientation to the threat rather than avoidance [23]. For example, changing the negative view of traumatic events, changing the evaluation of self in trauma, and affirming one’s own strength is highly self-affirming, which can reduce injury and effectively promote PTG. Self-affirmations can reassure people that they have integrity and that life is adequate despite adversities [23]. Personal adequacy also plays a role during traumatic events, promoting greater self-affirmation and contributing to coping with future traumatic experiences.

Deliberate rumination refers to the adaptive cognitive process of paying attention to the negative emotions or experiences caused by traumatic events and actively, consciously, and purposefully explaining traumatic events, seeking meaning, and exploring feelings [24]. Studies have shown that individuals’ deliberate rumination is positively correlated with their resilience and PTG [15]. Our findings also indicated that nurses’ experiences of deliberate rumination helped them grow in the face of adversity. In traditional Chinese culture, being reserved and thoughtful is considered desirable, and people believe that those with more introverted personalities tend to have a calm approach and strong ability to deal with things. Deliberate rumination is similar to such a personality type, emphasizing deep inner thinking. Simultaneously, some studies have shown that the ability to ruminate is related to work experience [25]. The participants in our study had a long overall working tenure, and they used different methods of deliberate rumination (e.g. psychological adjustment, downward comparison), which effectively promoted the realization of PTG.

Cognitive restructuring is another important promoting factor of PTG. Nurses could reasonably transform negative events through CR and transform unfavourable conditions into favourable ones [16]. As described by the participants, CR is a kind of re-cognition and transformation of personal mentality, environment, and the meaning of traumatic events under individuals’ subjective will. According to self-determination theory, when certain needs are disrupted after a traumatic event, individuals will still act to restore themselves to a state of well-being [26]. Thus, traumatic events are more of a catalyst for individuals to restructure in a non-adaptive state, revealing their previously unexposed potential, such as dealing with problems, learning from the environment, and adjusting their mindset. Nurses gained a new life concept through CR, that is, a kind of PTG. They acquired more experience and a better mindset to reflect on and face future traumatic events.

Internal factors influence the trend of individual PTG, and the internal strength of nurses should be fully guided and stimulated in nursing management and education. This includes highlighting the importance of psychological capital, rumination and CR ability, professional confidence, and other aspects in courses or training. A positive coping style alleviates negative emotions and helps to achieve PTG by improving the individuals’ understanding of negative events and enhancing their ability to solve and cope with problems, which often depends on their self-efficacy and execution. This ability bolsters their confidence in finding solutions. A proactive problem-solving approach is a good guide in the clinical work environment. Nurse leaders should focus on the cultivation of team action and problem-solving character. Resilience implies the ability to bounce back or easily recover when confronted by adversity, trauma, misfortune, or change [27]. Researchers defined resilience in nursing as a measure of a nurse’s ability to cope with stressors and mental health threats, contending that resilient people are emotionally calmer when dealing with catastrophic situations. Resilience has a significant positive effect on PTG [28], and it is particularly important for nursing staff to cultivate psychological resilience.

External factors were the second theme to emerge from this study, which contained factors that were influenced by others or the environment to promote nurses’ PTG. Social support stemmed from the government, organization, colleagues, and family. In our study, nurses appreciated receiving material or spiritual support under adverse circumstances. Studies have reported that for medical staff, social support has a direct positive predictive effect on personal PTG and can also indirectly promote PTG through resilience [29]. Simultaneously, many studies have shown that social support positively promotes the work satisfaction, career dedication, well-being, and mental health of nurses [30, 31]. Therefore, it is necessary to improve workplace social support, especially in stressful work environments that are prone to trauma exposure [17]. Organizations and departments should focus on the practical needs of nurses and can implement measures such as improving welfare, strengthening care, improving the working environment, and holding relaxation activities. It is also necessary to maintain unity and a harmonious atmosphere within the organization. Stronger team cohesion can help establish strong organizational support to reduce the impact of trauma.

Among the promoting factors of PTG of nurses, transformational leadership emphasizes the behavioural style of nursing leaders and the subjective feelings of nurses. After traumatic events, nursing leaders should pay more attention to the psychological state of nurses in daily work and actively communicate with nurses. Active listening is a practical strategy that can help nurses to obtain psychological support, and nurse leaders should encourage nurses to express their emotions and listen to and address nurses’ concerns and comments promptly. In addition, negative psychological conditions caused by overwork or unfair treatment should be avoided. A reasonable working system and the scientific working process should be formulated according to workload and personnel allocation ratio.

Role modelling helps nurses to build confidence and realize the possibility of overcoming difficulties. COVID-19 is a global public health emergency, and the sense of duty and honour of medical staff attract the public’s attention. On one hand, as role models, professional mentors, clinical experts, and senior colleagues have richer PTG experience and can guide and help nurses to manage the damage caused by trauma. On the other hand, their professionalism and dedication will better inspire nurses to establish correct professional values and strengthen their self-worth. Thus, it is necessary to strengthen the publicity of excellent role models or hold face-to-face symposia to enhance their guiding roles.

Studies have identified a significant internal relationship between nurses’ turnover intention and lack of post-traumatic support [32]. Nursing managers should consider how to implement external strategies to help nurses achieve PTG, which is related to the construction of nursing teams and the quality of clinical nursing. Various forms of education and ability training are encouraged; for example, mindfulness workshops, situational simulation exercise, peer support, and other methods can be used to train nurses to better cope with traumatic events. Ongoing education and guidance can protect traumatized nurses from absorbing or internalizing unmanageable emotions, which may lead to compassion fatigue. This can help them to recover more quickly after experiencing trauma and promote career growth. Team resilience is a protective factor for individual resilience, manifested as the collective psychological state of team members’ shared cognition, motivation, or emotions [33]. When team adversity is perceived, team members invoke their positive psychological resources and extend individual resilience to the team level through interpersonal interactions [12]. Taking measures to improve team resilience is effective in promoting individual PTG, including simulation education and team management of clinical aggression training, multimodal resilience training program, stress management and resilience training, and so forth. Finally, it is hoped that a special support organization for the mental health of medical staff should be established in various medical institutions to form a trauma-informed culture, which will be conducive to implementing team PTG intervention strategies.

The current study had several limitations. First, this was a qualitative study, and the results are not replicable because the participants, their experiences, and the contexts were all unique. It may lead to biased results. Moreover, the study was investigated in a single clinical setting, which may have hindered the generalizability of the results. Replicating this study in different clinical settings and among larger samples and samples from different cultures could help produce more applicable results. For example, future research could incorporate insights from other medical professionals or conduct research globally.

## Conclusions

The promoting factors of PTG of emergency nurses originated from different sources such as individuals, organizations, and society. However, there were many ways to improve the promotion of PTG. Specifically, in addition to good psychological adjustment of the individual, society, hospitals, and nursing managers should focus on establishing supportive PTG strategies to circumvent the lack of individual coping ability. This includes various forms of education and capacity-building, such as mindfulness workshops, situational simulation exercise, peer support, and other methods. Organizations and departments should pay attention to the practical needs of nurses, the ultimate purpose is to improve the retention rate and career growth of nurses, similarly, these interventions are applicable to other medical staff for posttraumatic psychological rehabilitation.

## Data Availability

The original contributions presented in the study are included in the article, further inquiries can be directed to the corresponding authors.

## Abbreviations

COVID-19: 2019 Coronavirus disease
PTG: Post-traumatic growth
PTSD: Post-traumatic stress disorder
SARS-CoV-2: Severe acute respiratory syndrome coronavirus 2
CR: Cognitive Restructuring

## References

[1] World Health Organization. 2020. Coronavirus disease (COVID-19). https://covid19.who.int/. Accessed 8 Sept 2022.

[2] Shanghai Municipal Health Commission. 2022. https://wsjkw.sh.gov.cn/. Accessed 8 Sept 2022.

[3] People’s Daily Online. 2022. More than 38,000 medical workers from 15 provinces rushed to Shanghai. http://sx.people.com.cn/n2/2022/0405/c352664-35208453.html. Accessed 10 Sept 2022.

[4] Sohrabi C, Alsafi Z, O’Neill N, Kerwan A, Al-Jabir A, Iosifidis C, Agha R (2020). World Health Organization declares global emergency: a review of the 2019 novel coronavirus (COVID-19) [published correction appears in Int J Surg. 2020;77:217]. Int J Surg.

[5] Bazazan A, Dianat I, Mombeini Z, Aynehchi A, Jafarabadi MA (2019). Fatigue as a mediator of the relationship between quality of life and mental health problems in hospital nurses. Accid Anal Prev.

[6] Foli KJ, Reddick B, Zhang L, Krcelich K (2020). Nurses’ psychological trauma: ‘They leave me lying awake at night’. Arch Psychiatr Nurs.

[7] Foli KJ, Forster A, Cheng C, Zhang L, Chiu YC (2021). Voices from the COVID-19 frontline: nurses’ trauma and coping. J Adv Nurs.

[8] McLennan J, Evans L, Cowlishaw S, Pamment L, Wright L (2016). Secondary traumatic stress in postdisaster field research interviewers. J Trauma Stress.

[9] Tedeschi RG, Calhoun LG (1996). The Posttraumatic Growth Inventory: measuring the positive legacy of trauma. J Trauma Stress.

[10] Sandelowski M (2010). What’s in a name? Qualitative description revisited. Res Nurs Health.

[11] Palinkas LA, Horwitz SM, Green CA, Wisdom JP, Duan N, Hoagwood K (2015). Purposeful sampling for qualitative data collection and analysis in mixed method implementation research. Adm Policy Ment Health.

[12] Jiang J, Liu Y, Han P, Zhang P, Shao H, Peng H, Duan X (2022). Psychological resilience of emergency nurses during COVID-19 epidemic in Shanghai: a qualitative study. Front Public Health.

[13] Jiang J, Han P, Huang X, Liu Y, Shao H, Zeng L, Duan X (2022). Post-traumatic growth experience of first-line emergency nurses infected with COVID-19 during the epidemic period-A qualitative study in Shanghai, China. Front Public Health.

[14] Morrow R, Rodriguez A, King N (2015). Colaizzi’s descriptive phenomenological method. Psychologist.

[15] Xu W, Jiang H, Zhou Y, Zhou L, Fu H (2019). Intrusive rumination, deliberate rumination, and posttraumatic growth among adolescents after a tornado: the role of social support. J Nerv Ment Dis.

[16] Wenzel A (2017). Basic strategies of cognitive behavioral therapy. Psychiatr Clin North Am.

[17] Jiang J, Han P, Huang X, Liu Y, Shao H, Zeng L, Duan X. Post-traumatic growth experience of first-line emergency nurses infected with COVID-19 during the epidemic period -- A qualitative study in Shanghai, China. Front Public Health. 3808. doi:10.3389/fpubh.2022.1015316. 10.3389/fpubh.2022.1015316 PMC9597244 36311593

[18] Poghosyan L, Bernhardt J (2018). Transformational leadership to promote nurse practitioner practice in primary care. J Nurs Manag.

[19] Tomaszek K, Muchacka-Cymerman A (2020). Thinking about my existence during COVID-19, I feel anxiety and awe–the mediating role of existential anxiety and life satisfaction on the relationship between PTSD symptoms and post-traumatic growth. Int J Environ Res Public Health.

[20] Cui PP, Wang PP, Wang K, Ping Z, Wang P, Chen C (2021). Post-traumatic growth and influencing factors among frontline nurses fighting against COVID-19. Occup Environ Med.

[21] Han SJ, Chun JY, Bae HJ (2022). Post-traumatic growth of nurses in COVID-19 designated Hospitals in Korea. Int J Environ Res Public Health.

[22] Bryngeirsdottir HS, Halldorsdottir S (2022). The challenging journey from trauma to post-traumatic growth: lived experiences of facilitating and hindering factors. Scand J Caring Sci.

[23] Cohen GL, Sherman DK (2014). The psychology of change: self-affirmation and social psychological intervention. Annu Rev Psychol.

[24] Cárdenas Castro M, Arnoso Martínez M, Faúndez Abarca X (2019). Deliberate rumination and positive reappraisal as serial mediators between life impact and posttraumatic growth in victims of state terrorism in Chile (1973–1990). J Interpers Violence.

[25] Chang AK, Yoon H, Jang JH (2021). Predictors of posttraumatic growth of intensive care unit nurses in Korea. Jpn J Nurs Sci.

[26] Patrick H, Williams GC (2012). Self-determination theory: its application to health behavior and complementarity with motivational interviewing. Int J Behav Nutr Phys Act.

[27] Dyer JG, McGuinness TM (1996). Resilience: analysis of the concept. Arch Psychiatr Nurs.

[28] Tranter H, Brooks M, Khan R (2021). Emotional resilience and event centrality mediate posttraumatic growth following adverse childhood experiences. Psychol Trauma.

[29] Yeo HJ, Park HS (2020). The structural analysis of variables related to posttraumatic growth among psychiatric nurses. J Korean Acad Nurs.

[30] Labrague LJ, De Los Santos JAA (2020). COVID-19 anxiety among front-line nurses: predictive role of organisational support, personal resilience and social support. J Nurs Manag.

[31] Sun N, Wei L, Shi S, Jiao D, Song R, Ma L, Wang H, Wang C, Wang Z, You Y, Liu S (2020). A qualitative study on the psychological experience of caregivers of COVID-19 patients. Am J Infect Control.

[32] Crowe S, Howard AF, Vanderspank B (2022). The mental health impact of the COVID-19 pandemic on canadian critical care nurses. Intensive Crit Care Nurs.

[33] Morgan PB, Fletcher D, Sarkar M (2017). Recent developments in team resilience research in elite sport. Curr Opin Psychol.

